# Effect of Different Collagen on Anterior Cruciate Ligament Transection and Medial Meniscectomy-Induced Osteoarthritis Male Rats

**DOI:** 10.3389/fbioe.2022.917474

**Published:** 2022-07-05

**Authors:** Jerrell Felim, Chun-Kai Chen, David Tsou, Hsiang-Ping Kuo, Zwe-Ling Kong

**Affiliations:** ^1^ Laboratory of Cellular Immunology, Department of Food Science, National Taiwan Ocean University, Keelung City, Taiwan; ^2^ Biotaiwan Foundation, New Taipei City, Taiwan

**Keywords:** osteoarthritis, *Rhopilema esculentum*, jellyfish, collagen, collagen peptide, glycine

## Abstract

Osteoarthritis (OA) is a common type of arthritis characterized by degeneration of the articular cartilage and joint dysfunction. Various pharmacological and non-pharmacological techniques have been used to manage these diseases. Due to the diverse therapeutic properties of marine collagen, it has received considerable attention in its pharmacological application. Thus, the purpose of this study was to compare the efficacy of jellyfish collagen, collagen peptide, other sources of marine collagen, and glycine in treating OA. In the OA rat model, an anterior cruciate ligament transection combined with medial meniscectomy surgery (ACLT + MMx) was used to induce osteoarthritis in rats. Two weeks before surgery, male Sprague–Dawley rats were fed a chow-fat diet. After 6 weeks of treatment with collagen, collagen peptide, and glycine, the results show that they could inhibit the production of proinflammatory cytokines and their derivatives, such as COX-2, MMP-13, and CTX-II levels; therefore, it can attenuate cartilage degradation. Moreover, collagen peptides can promote the synthesis of collagen type II in cartilage. These results demonstrate that collagen and glycine have been shown to have protective properties against OA cartilage degradation. In contrast, collagen peptides have been shown to show cartilage regeneration but less protective properties. Jellyfish collagen peptide at a dose of 5 mg/kg b. w. has the most significant potential for treating OA because it protects and regenerates cartilage in the knee.

## Graphical Abstract

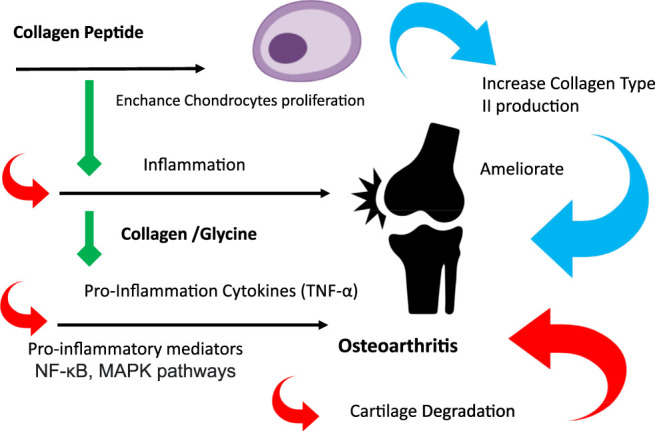

## 1 Introduction

Osteoarthritis (OA), the most common form of arthritis, is generally characterized by slowly progressive degeneration of articular cartilage, particularly in weight-bearing joints (Sabatini et al., 2004). The complex network of type II collagen, proteoglycans, and accessory proteins such as fibronectin maintains the integrity of cartilage tissue. Chondrocytes synthesize and integrate these molecules into the residual extracellular matrix (ECM). The loss of ECM in cartilage is associated with an increase in type II collagen cleavage by collagenase, aggrecan cleavage, and proteoglycan degradation (Felson et al., 2000). The progressive imbalance between matrix degradation and regeneration results in significant decrease in the amount of type II collagen, which eventually causes cartilage damage (Rizkalla et al., 1992; Hollander et al., 1995; Nelson et al., 1998; Aigner and Stove, 2003).

Most current treatment modes aim to alleviate pain and reduce inflammation, thereby improving function and reducing disability with fewer adverse effects (Lindler et al., 2020). A more natural approach utilizing nutritional supplements with a higher level of safety and effectiveness has garnered considerable interest. Nutritional supplements are more likely to provide long-term health benefits by their very nature. Collagen is a necessary component of the pharmaceutical and food industries. Collagen is a multifunctional macro protein that accounts for approximately 20%–30% of all proteins found in living organisms (Lee et al., 2001; Addad et al., 2011) and represents the main structural component of the extracellular matrix in all connective tissues. Studies have demonstrated that collagen effectively improves OA symptoms by decreasing both the total WOMAC index and VAS score (García-Coronado et al., 2019).

Collagen-derived peptides are functional peptides with a variety of physiological functions. Collagen peptide is absorbed and distributed into joint tissues, where it acts as an analgesic and anti-inflammatory (Zdzieblik et al., 2021). Collagen peptides are defined by high concentrations of the amino acids glycine (Gly), hydroxyproline (Hyp), and proline (Pro). Numerous studies have demonstrated that collagen hydrolyzate can enhance chondrocyte biosynthesis of type II collagen *in vitro*, suggesting that it may be used to treat osteoarthritis (Oesser et al., 1999; Ameye and Chee, 2006; Bello and Oesser, 2006; Mcalindon et al., 2011).

Glycine, proline, and lysine each play a unique role in collagen structure, and their insufficient availability may contribute to the difficulty of collagen synthesis and regeneration (De Paz-Lugo et al., 2018). Glycine, which comprises 33% of collagen residues, has traditionally been classified as a ‘non-essential’ amino acid since it can be synthesized by human metabolism from serine (Bowes and Kenten, 1948; Pauling and Corey, 1951). However, Melendez-Hevia and Paz-Lugo (2008) demonstrated that the glycine synthesis pathway has a strong stoichiometric restriction that limits its production regardless of the capacity and regulatory mechanisms of the enzymes. Because its synthesis capacity is significantly less than its actual requirement, glycine must be considered an essential amino acid. The effects of glycine in inhibiting proinflammatory cytokine expression have been studied *in vitro* and confirmed in different animal models, especially for beneficial effects in reducing pain in OA of the hip and knee (Li et al., 2001; Mastbergen et al., 2007; Hartog et al., 2013).

Structure, self-association, and ligand binding, all of which are essential to collagen’s physiological functions, have been elucidated by a variety of physicochemical techniques applied to its study (Brodsky and Persikov, 2005). Native collagen properties are very different from those of hydrolyzed collagen. Its solubility and functional activity (antioxidant and antimicrobial) are related to the type and degree of hydrolysis and the type of enzyme used in the process (León-López et al., 2019). Hydrolysis affects not only the size but also the physicochemical and biological properties of peptides (Zhang et al., 2017). Collagen composition and degree of hydrolysis affect its functional properties such as antioxidant capacity, antimicrobial activity, and bioavailability. These characteristics are primarily related to the molecular weight value (Wang et al., 2018).

The jellyfish umbrella is rich in collagenous protein (Nagai et al., 1999) and can be considered a potential collagen source. The jellyfish, *Rhopilema esculentum*, is a high-yield aquatic animal in China. It has been used as Chinese food for high nutrition values and pharmacological activities for thousands of years and has recently become popular in other countries (Yusuf et al., 2018; Leone et al., 2019; Duarte et al., 2021). Li et al., (2014) also reported that proteins derived from jellyfish *Rhopilema esculentum* have antioxidant activity and could be utilized in the food and pharmaceutical industries. OA needs to look for a new treatment to manage this disease, according to the explanation.

*Ganoderma lucidum*, a fungus known by its many names such as “Reishi,” “Ling Zhi,” and “Mannentake,” for hundreds or even thousands of years, is recognized as powerful medicinal fungi because it has properties often associated with health and healing, long life, knowledge, and happiness (Valverde et al., 2015). In addition, the glutamic acid protease from the peptidase G1 family of the *Ganoderma lucidum* was found as a major protein (Kumakura et al., 2019). PepG1 demonstrated peptidase activity toward collagen, identified as collagenases, and pepG1 was discovered to be a very stable protein for both pH and temperature stability (Jensen et al., 2010; Ahmed et al., 2018). Therefore, fermentation with *Ganoderma lucidum* was chosen to hydrolyze collagen from *Rhopilema esculentum.*

This research aims to investigate the effect of *Rhopilema esculentum* collagen, *Rhopilema esculentum* collagen peptide, collagen peptide from other marine sources, and glycine on anterior cruciate ligament transection (ACLT) and medial meniscectomy (MMx)–induced osteoarthritis in Sprague–Dawley rats.

## 2 Materials and Methods

### 2.1 Material

*Rhopilema esculentum* was obtained from Fuzhou Jellyfish Investment Co., Ltd. Seventy male Sprague–Dawley rats (10-weeks old) were purchased from BioLASCO Co. Ltd. (Yilan, Taiwan). Glucosamine sulfate (GS) was purchased from Viatril-S (United Kingdom). Glycine was brought from Yeongca Co., Ltd. Tilapia peptide (IXOS HDL-50SP) was obtained from Toong Yeuan Enterprise Co., Ltd. Matrix™ Neo was a kind gift from Kanematsu Chemical Corp, Collactive™ was obtained from Green Strong International Co., Ltd. The standard laboratory chow-fed diet (Laboratory Rodent Diet 5001) was purchased from PMI Nutrition International, Inc. (Brentwood, MO, United States). Matrix metalloproteinase-13 (MMP-13), prostaglandin E2, C-Terminal Cross-Linked Telopeptide of Type II Collagen, and Tumor Nuclear Factor Alpha ELISA kits were purchased from Finetest biotechnology Inc. (Wuhan, China). Zoletil 50 was purchased from Virbac (Carros, France). Lofalin injections (cefazolin sodium) were purchased from Gentle Pharm Co. Ltd. (Yunlin, Taiwan). Formaldehyde solution was purchased from Avantor Performance Materials Inc. (Radnor, PA, United States).

### 2.2 *Rhopilema esculentum* Extraction

#### 2.2.1 *Rhopilema esculentum* Collagen Extraction

*Rhopilema esculentum* collagen extraction was performed according to previously used methods (Nagai et al., 1999), with modification. *Rhopilema esculentum* was digested by 0.5 M acetic acid containing 10,000 U/g papain for 24 h at 4°C. The papain-soluble collagen liquid was centrifuged at 10,500 rpm for 1 hour, and the supernatant was salted out by adding NaCl to a final concentration of 4.5 M. The resultant precipitate was obtained by centrifugation at 10,500 rpm for 1 hour and dissolved in 0.5 M acetic acid, dialyzed against double-distilled water, and lyophilized. *Rhopilema esculentum* collagen was transferred into fresh bottles and dried using a freeze dryer to obtain the final jellyfish collagen (JC).

#### 2.2.2 *Rhopilema esculentum* Collagen Peptide

Collagen peptides from *Rhopilema esculentum* were extracted using previously described methods (Nagai et al., 1999) with modification. Jellyfish were first pretreated for 24 h with NaOH 0.1 M to eliminate non-collagenous protein, followed by neutralization with acetic acid 0.5 M. The jellyfish were then fermented for 14 days with 5% *Ganoderma lucidum* strain at 25°C, 120 rpm, 2% soybean +2% sugar +0.5 percent yeast extract (done by Grape King Co. Ltd). After discarding the jellyfish, the collagen peptide is freeze-dried to obtain the final jellyfish collagen peptide (JCP).

#### 2.2.3 General Analysis of *Rhopilema esculentum* and Collagen Peptide

Proximate analysis was carried out following the standard of AOAC (Horwitz and Latimer, 2000) for moisture, protein, crude carbohydrate, ash, and lipid content. DPPH antioxidant assay was performed according to Nicklisch and Waite (2014). DPPH solutions were prepared in ethanol. Solvents and other chemicals used in this experiment were of analytical grade. The reaction tubes were wrapped in aluminum foil in triplicate and kept at 30°C for 30 min in the dark. All measurements were taken in low-light conditions. At 517 nm, spectrophotometric measurements were taken. FT-IR spectra of lyophilized samples were measured on an FT-IR spectrometer using the method described by previous studies (Wang et al., 2018). The spectra were generated using a single scan with a wavelength range of 4000 to 400 cm^−1^ and a resolution of 1 cm^−1^.

### 2.3 Animal and Diets

Ten-week-old male Sprague–Dawley rats were purchased from the National Laboratory Animal Center (Yilan, Taiwan). All rats were fed a standard chow-fed diet (Laboratory Rodent Diet 5001) and water *ad libitum*. The rats were acclimatized for 2 weeks and were housed one rat per cage in a room maintained at 25 ± 2°C under a 12 h day/night cycle throughout experimentation. The Institutional Animal Care and Use Committee of the National Taiwan Ocean University reviewed and approved all protocols. The rats were fed for 6 weeks accordingly. They were subdivided into ten groups (n = 7 rats/group): the control group (surgery with an opened knee-joint capsule without ACLT + MMx) and nine groups receiving surgery with ACLT + MMx.

Seven groups of the OA groups were administered daily oral gavage with Jellyfish Collagen of 1 mg/kg b. w. and Jellyfish collagen peptide with two different doses of JCP with 1 mg/kg b. w. and 5 mg/kg b. w. For comparison of different collagen peptides, we use tilapia collagen peptide at 1 mg/kg b. w., collactive collagen peptide at 1 mg/kg b. w., and matrix collagen at 1 mg/kg b. w., in comparison to glycine at 1 mg/kg b. w, and the last group was given a dose of glucosamine sulfate (GS) as a positive control (100 mg/kg body weight; GS). The dose of JCP was evaluated from some previous studies related to oral administration of jellyfish collagen and jellyfish collagen peptide in rats (Di Cesare Mannelli et al., 2013). The dose of GS was considered according to previous studies (Wen et al., 2010). The rats were euthanized by exposure to carbon dioxide (CO_2_) in an empty chamber at week 9 of the experiment (treatment with samples or GS for 6 weeks). The rats were starved for 12 h before surgery and euthanization, and on the day of euthanization, the body weight was measured using a weighing scale. Whole blood and knee joints were collected and stored for further analysis. The whole blood of rats was collected from the abdominal aorta of the rats on the day of euthanization using a heparinized syringe. The plasma or serum was separated from whole blood by centrifugation (Kubota Centrifuge 3500; Kubota Corp., Tokyo, Japan) at 3,000 rpm and 4°C for 15 min. The supernatant was gently collected by using a micropipette and stored at −20°C for future analysis (Mussbacher et al., 2017).

#### 2.3.1 Surgery-Induced Osteoarthritis

According to previous methods, the ACLT + MMx surgery to induce OA was performed (Hayami et al., 2006). Briefly, rats were anesthetized by intraperitoneal injection of zoletil (25 mg/kg body weight), and the hair on the right knee of rats was shaved using a digital hair clipper. In control, surgery was performed by opening the knee-joint capsule without ACLT + MMx, whereas the OA groups underwent ACLT + MMx. The right knee joint was exposed using a medial parapatellar approach, then the patella was laterally dislocated, and the knee was extended fully, followed by ACL transection using micro scissors, and medial meniscus was resected. The knee-joint capsule and skin were closed by sewing with chromic catgut sterile (4-0) and silk braided sterile sutures (3-0; Unik Surgical Sutures Mfg, Co. Ltd, Taipei, Taiwan), respectively. The rats were then intraperitoneally injected with cefazolin antibiotic (30 mg/kg/day) for 3 days after surgery to prevent surgery-related infection.

### Biochemical Assays and Histological Analysis

The serum tumor necrosis factor-alpha, C-telopeptide fragments of type II collagen Matrix metalloproteinase-13, and cyclooxygenase-2 were measured following Finetest’s protocols. According to Yoon et al. (2007), an aliquot of the incubation blood plasma (100 μL) was transferred to 96-well plates. Nitrite was determined spectrophotometrically using the Griess reagent (0.8% sulfanilamide, 0.75% N-(naphthyethylene) diamine in 0.5 N HCl) by mixing 100 μL of the Griess reagent. After 15 min incubation at room temperature, the nitrite concentrations were measured at 540 nm using a microplate reader. Sodium nitrate (0.5–100 μM) was used as the nitrite standard, and nitrite was linear over this concentration range. According to Cardiff et al., (2014), H&E staining was performed by placing the glass slides that hold the paraffin sections in staining racks. The paraffin from the samples in three changes of xylene was cleared for 2 min per change. The slides were transferred through three changes of 100% ethanol for 2 min per change, then they were transferred to 95% ethanol for 2 min, and then transferred to 70% ethanol for 2 min. The slides were rinsed in running tap water at room temperature for at least 2 min. The samples were stained in hematoxylin solution for 3 min and the slides were placed under running tap water at room temperature for at least 5 min. The samples were stained in working eosin Y solution for 2 min and the slides were dipped in 95% ethanol about 20 times. They were transferred to 95% ethanol for 2 min and transferred through two changes of 100% ethanol for 2 min per change. The samples were cleared in three changes of xylene for 2 min per change. A drop of permount is placed over the tissue on each slide and a coverslip is added to it. The slides are viewed using a microscope. According to (Schmitz et al., 2010), Safranin-O staining was performed by deparaffinizing and hydrating the slide to distilled water and stained with Weigert’s hematoxylin working solution for 10 min. They were washed in running tap water for 10 min, stained with fast green (FCF) solution for 5 min, rinsed quickly with a 1% acetic acid solution for no more than 10–15 s, stained in 0.1% safranin O solution for 5 min, dehydrated and cleared with absolute ethyl alcohol for 5 min three times and xylene for 5 min, and mounted with resinous mounting media.

### 2.5 Statistical Analysis

All data were expressed as the mean ± standard deviation (SD). The experimental data were analyzed by Statistical Package software such as Statistical Product and Services Solution (SPSS 22.0, IBM Corporation, New York, United States) and GraphPad Prism 5 for statistical analysis of variance (ANOVA). Duncan’s multiple range test was carried out to analyze the significant difference; *p* < 0.05 was considered significantly different.

## 3 Results

### 3.1 General Analysis of *Rhopilema esculentum* and Collagen Peptide

Proximate analysis from jellyfish bell shows that ash content was observed at about 72.88% dry weight, followed by protein with 13.93%, carbohydrate with 12.47%, and lipid with 0.71%, with a water content of 74.59% (Table 1). The experimental results showed that the jellyfish’s collagen wet weight was 0.495 g/100 g wet weight (Table 2). Collagen extraction yield on dry weight equals 1.948 g/100-g dry weight.

**TABLE 1:** Proximate composition of jellyfish.

Component	Wet weight (%)	Dry weight (%)
Moisture	74.59 ± 0.1100	—
Crude protein	3.54 ± 0.0008	13.93 ± 0.0039
Crude lipid	0.18 ± 0.0060	0.71 ± 0.0200
Ash	18.52 ± 0.0100	72.88 ± 0.0400
Carbohydrates	3.17	12.47

**TABLE 2:** Collagen yield.

	Extract yield (g/100 g)
Jellyfish	100
Jellyfish collagen (Powder)	0.495

The IC_50_ value is a widely used parameter for determining the antioxidant activity of test samples. It is calculated as the antioxidant concentration required for reducing the initial DPPH concentration by 50% (Sánchez-Moreno et al., 1998). Lower IC_50_ shows higher antioxidant activity (Molyneux, 2004). In this study, IC_50_ values of *Rhopilema esculentum* collagen were 10.429 mg/ml, that for *Rhopilema esculentum* collagen peptide was 2.280 mg/ml (Table 3), IC_50_ of Tilapia collagen was 10.533 mg/ml, that of Collactive collagen peptide was 4.578, and that of matrix collagen was 7.266 mg/ml. Last, glycine shows IC_50_ of 22.832 mg/ml.

**TABLE 3:** DPPH radical scavenging effect of jellyfish collagen (JC) jellyfish collagen peptide (JCP) company “T” tilapia peptide, “collactive” collagen peptide, “matrix” collagen peptide, and glycine.

	The DPPH radical scavenging IC_50_ (mg/ml)
Jellyfish collagen	10.429
Jellyfish collagen peptide	2.280
Tilapia collagen peptide	10.533
Collactive collagen peptide	4.578
Matrix collagen peptide	7.266
Glycine	22.832

**FIGURE 1:**
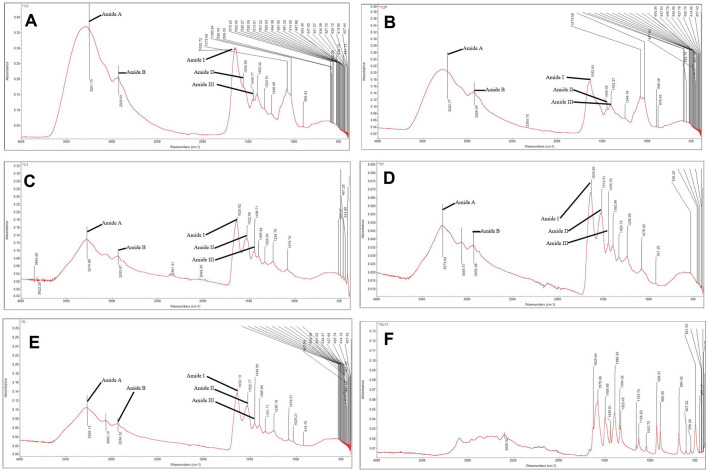
FTIR of **(A)** Rhopilema esculentum collagen, **(B)** Rhopilema esculentum collagen, **(C)** Tilapia Collagen Peptide, **(D)** Collactive, **(E)** Matrix Collagen Peptide, **(F)** Glycine.

Infrared spectroscopy is sensitive to the chemical structures of molecules and can be used to find out how proteins and polypeptides differ in various states, concentrations, and environments. It is also a useful tool for finding out how proteins and polypeptides fold into their secondary structures (Yang et al., 2016). Collagen extracted from the jellyfish *Rhopilema esculentum* exhibited characteristic amide A, B, and amide I, II, and III peaks in the FT-IR spectrum. 3251.7 cm^−1^ was found to be the band position of amide A. The band position of amide B was observed at ∼2929.81 cm-1 due to asymmetrical stretching of CH_2_ (Zhang et al., 2014). The amide I band of this collagen was detected at 1632.72 cm-1. The amide II band was observed at ∼1556.56 cm−1. At 1246.49 cm^−1^, the amide III peak was discovered, corresponding to C—H stretching vibrations (Figure 1A). The intensity of the amide peak is associated with the triple helical structure of collagen.

In the present study, as to collagen hydrolyzates, the positions of FT-IR bands were nearly unchanged after hydrolyses. The intensity of the amide I peak of *Rhopilema esculentum* collagen peptide, tilapia collagen peptide, collactive collagen peptide, and matrix collagen peptide was lower than that of *Rhopilema esculentum* collagens, and this indicated that some of the collagen was cut into polypeptides, which destroyed some of the helical structures of the collagen hydrolyzates (Figure 1B–F.) (Li et al., 2013). Hydrolyzed collagen samples presented peaks of medium intensity at lower wavelengths, of 800 to 1,200 cm-1. The region between 1200–800 cm-1 is referred to as the fingerprint region, where similar molecules can give different absorption patterns (Böcker et al., 2017; Kristoffersen et al., 2019; Wubshet et al., 2019; Måge et al., 2021).

### 3.2 Effects of Different Collagen on Osteoarthritis in Rats

#### 3.2.1 Body Weight and Organ Weight

**FIGURE 2:**
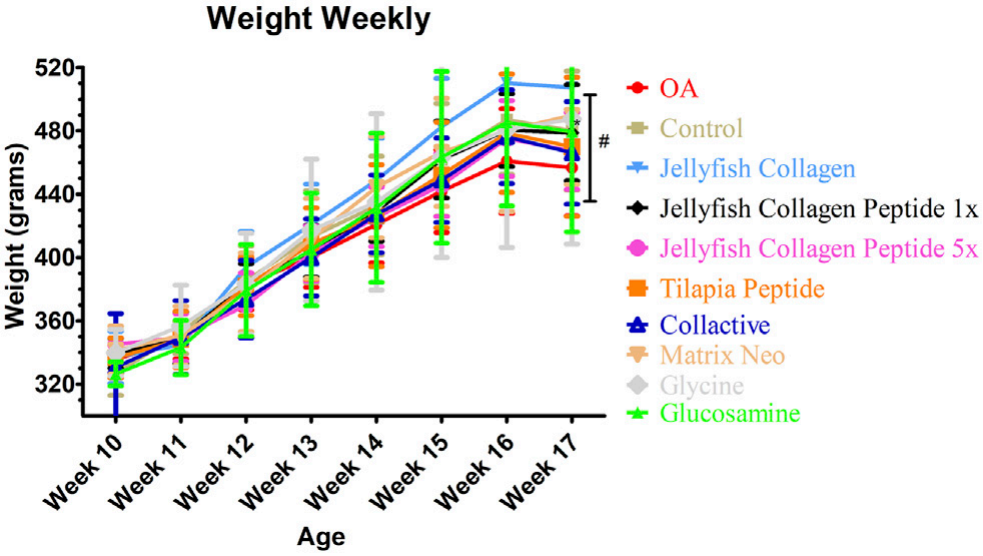
Average weekly weight of rats.

As illustrated in Figure 2, after sample treatment, the body weights of each group were not statistically different (*p* < 0.05). As shown in Table 4, there is an increase in liver weight in the OA group compared to the control group. Due to drug consumption, there is an increase in the weight of the liver. Still, a maximum of 13% of increasing weight in *Rhopilema esculentum* collagen peptide (5 mg/kg b. w.) was observed compared to the control group. There is no significant difference in the weight of the kidney, spleen, abdominal fat, and epididymis fat (*p* < 0.05).

**TABLE 4:** Mean weight of the rats’ organs (liver, spleen, kidney, abdominal fat, epididymis fat, etc). Values are presented as mean ± SD. Statistical analysis was performed using a one-way ANOVA test, followed by Duncan post-hoc. A group with different letters indicates a statistically significant difference, whereas those sharing the same letters do not show a statistically significant difference.

Rats/Organ	OA	Control	Jellyfish collagen	Jellyfish collagen peptide 1x	Jellyfish collagen peptide 5x	Tilapia collagen	Collactive	Matrix neo	Glycine	Glucosamine sulfate
Liver	3.4643 ± 0.1936 abc	3.2925 ± 0.1548 ab	3.6156 ± 0.4142 bc	3.4867 ± 0.1915 abc	3.7523 ± 0.4188c	3.5281 ± 0.3189 abc	3.3544 ± 0.1966 ab	3.5096 ± 0.2470 abc	3.2296 ± 0.4358 a	3.6225 ± 0.3279 bc
Spleen	0.1525 ± 0.0265 ab	0.1605 ± 0.0145 ab	0.1562 ± 0.0167 ab	0.1469 ± 0.0187 a	0.1606 ± 0.0194 ab	0.1591 ± 0.0148 ab	0.1537 ± 0.0163 ab	0.1440 ± 0.0192 a	0.1529 ± 0.0196 ab	0.1717 ± 0.0176 b
Kidney	0.8695 ± 0.0734 a	0.8498 ± 0.0804 a	0.8629 ± 0.0707 a	0.8774 ± 0.0652 a	0.9011 ± 0.1113 a	0.9028 ± 0.0565 a	0.8791 ± 0.0863 a	0.8417 ± 0.0757 a	0.8725 ± 0.1096 a	0.8836 ± 0.0771 a
Abdominal Fat	1.0646 ± 0.1077 a	1.3807 ± 0.2955 a	1.4076 ± 0.3548 a	1.2794 ± 0.4897 a	1.2899 ± 0.3299 a	1.1331 ± 0.5981 a	1.3661 ± 0.2038 a	1.5348 ± 0.4509 a	1.0564 ± 0.4481 a	1.4376 ± 0.6478 a
Epididymis Fat	1.0491 ± 0.3096 a	1.3205 ± 0.2259 a	1.5915 ± 0.5571 a	1.3116 ± 0.2925 a	1.1089 ± 0.2502 a	0.8949 ± 0.1357 a	1.1634 ± 0.2944 a	1.3371 ± 0.4444 a	0.9564 ± 0.4558 a	1.2850 ± 0.4760 a

#### 3.2.2 Tumor Nuclear Factor-Alpha, Nitric Oxide, and Cyclooxygenase Levels

**FIGURE 3:**
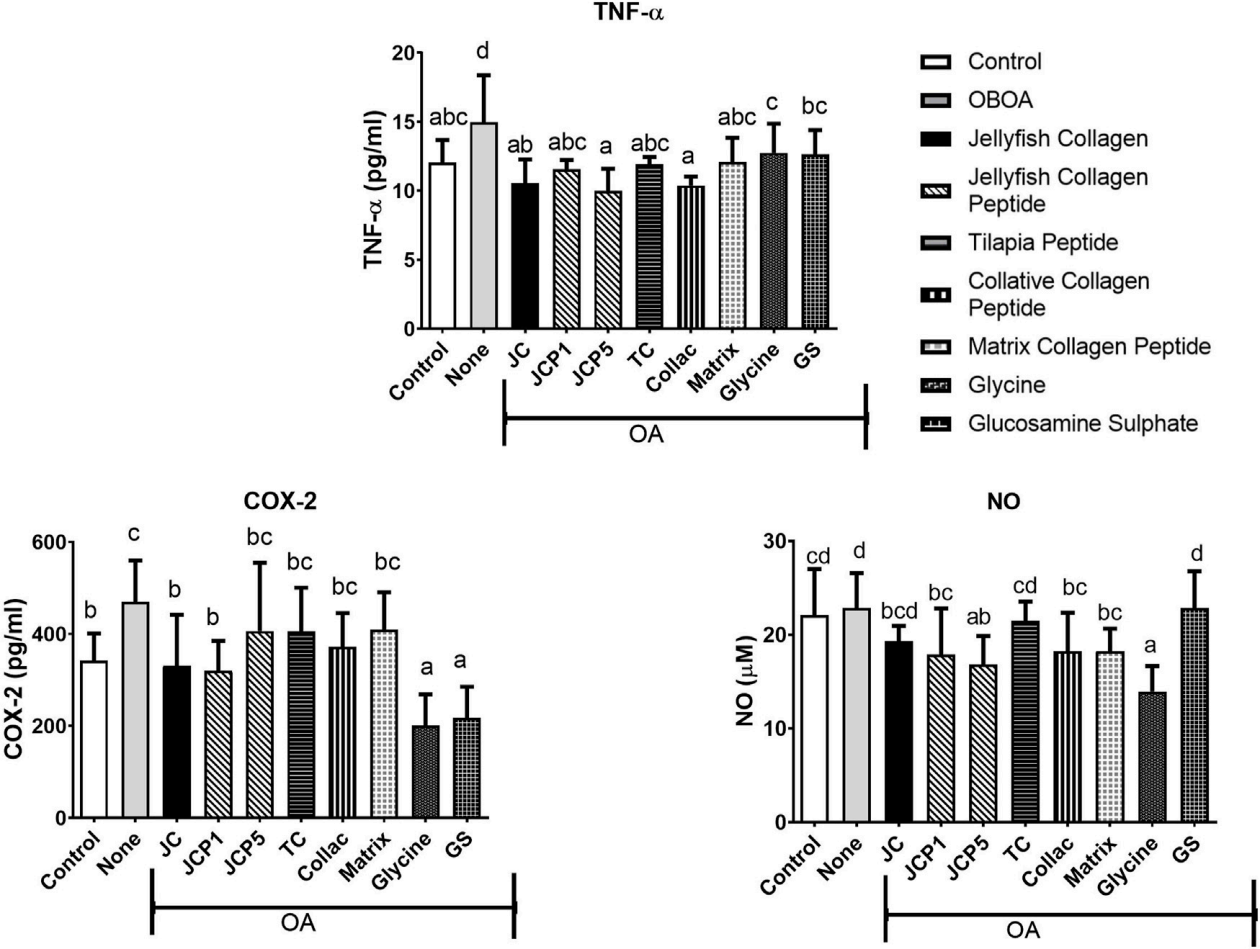
Tumour Necrosis Factor-alpha (TNF-α), Cyclooxygenase-2 (COX-2), and Nitric Oxide (NO) levels in rats' plasma.

Osteoarthritis-induced rats increased in TNF-α, COX-2, and NO levels, with levels of the OA group significantly higher than those of the control group (*p* < 0.05). However, TNF-α levels significantly decrease after treatment of jellyfish collagen, collagen peptide, and glycine compared to the OA group (*p* < 0.05) and show no significant difference from the control group (*p* < 0.05). In addition, COX-2 and NO levels were also significantly decreased in treated groups (*p* < 0.05). (Figure 3).

#### 3.2.3 Matrix Metallopeptidase 13 and C-Terminal Cross-Linked Telopeptide of Type II Collagen Levels

**FIGURE 4:**
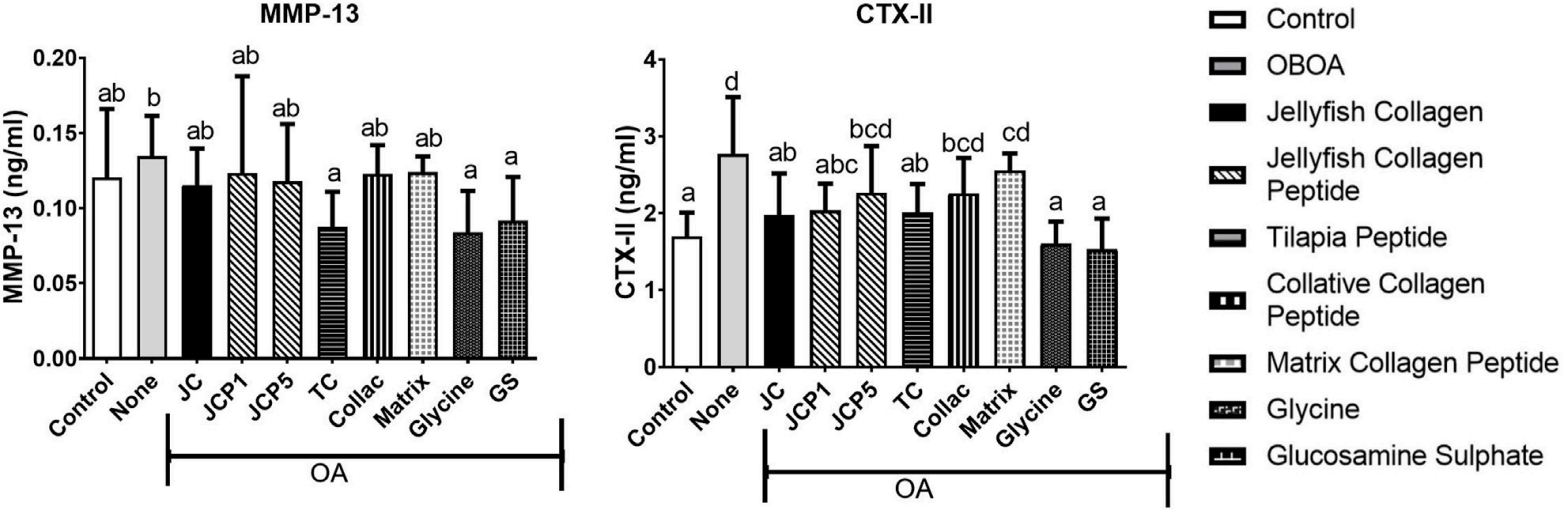
Matrix metallopeptidase 13 (MMP-13) and C-telopeptide fragments of type II collagen (CTX-II) levels in rats’ plasma.

The OA group shows elevated levels of MMP-13 and CTX-II (Figure 4), with CTX-II levels significantly higher than those of the control group. However, treatment with glycine and tilapia peptide is shown to decrease the MMP-13 (Figure 4) levels significantly (*p* < 0.05). Moreover, CTX-II levels also significantly decreased in the collagen and glycine group (*p* < 0.05) (Figure 4) compared to the OA group, showing higher protective effects of collagen and glycine.

#### 3.2.4 Histological Study

The articular cartilage in the control group was well-stained with Safranin O (which stains PG red) with no apparent loss of staining intensity, indicating that the matrix’s PG content was normal. The osteoarthritis group’s articular cartilage significantly reduced Safranin O staining intensity in the non-calcified region and a slight reduction in the calcified region, indicating a significant decrease in the matrix’s PG content.

**FIGURE 5:**
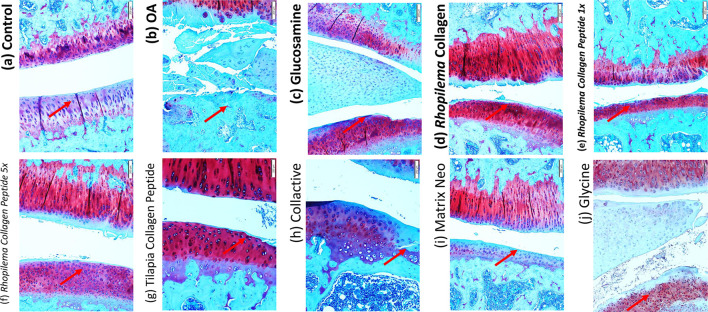
Histological analysis by Safranin O staining difference between the knee joint in ACLT+MMx surgery-induced OA male rats after treated with different samples.

In the osteoarthritis group, irregular notched surfaces, apparent reduction in cartilage thickness, chondrocytes shrunken with pyknotic nuclei, disorganized and few in number, and lost chondrocytes in some areas where tidemark was not clearly visible, and degenerative changes in the subchondral bone were all observed. The meniscus was severely frayed and torn, with disorganized collagen fibers (Figure 5A). After treatments with *Rhopilema* collagen, it showed a better histological appearance with a smoother surface. However, a notched surface was still observed, recovery of chondrocytes, and less degenerative change in subchondral bone, but it shows an unclear tide mark (Figure 5C). Compared to *Rhopilema* collagen peptide in the same dose, collagen shows a smoother surface and thicker cartilage. However, the collagen peptide shows a more apparent tide mark and fewer degenerative changes in the subchondral bone (Figure 5E). Moreover, the five-time dose of collagen peptide thickens the cartilage and has a non-calcified zone as in the control (Figure 5F).

Compared with the treatment with tilapia collagen peptide (Figure 5G), it shows protection for cartilage by a smoother surface of the treated group. The tide mark is also unclear between calcified and non-calcified zones, showing that chondrocyte regeneration is not perfectly carried out. The treatment with collactive shows a few irregular notches (Figure 5H). But, it shows a good chondrocyte pattern and an increase in number compared to the OA group. It shows there is recovery of cartilage with the regeneration of chondrocytes and collagen type II. Compared with collactive, matrix collagen peptide shows more irregular notches (Figure 5I). Treatment with glycine shows there is a smooth surface (Figure 5J) and recovered chondrocyte but shows a low level of the collagen type II produced. Safranin O–Fast Green Staining was performed.

#### 3.2.5 OARSI Scoring and Incapacitance Test

**FIGURE 6:**
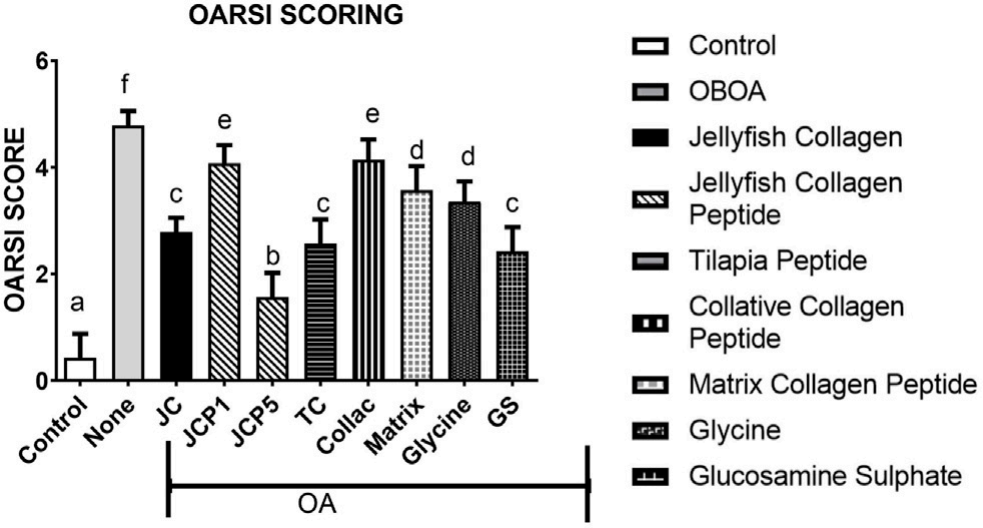
OARSI scoring of rats knee.

**FIGURE 7:**
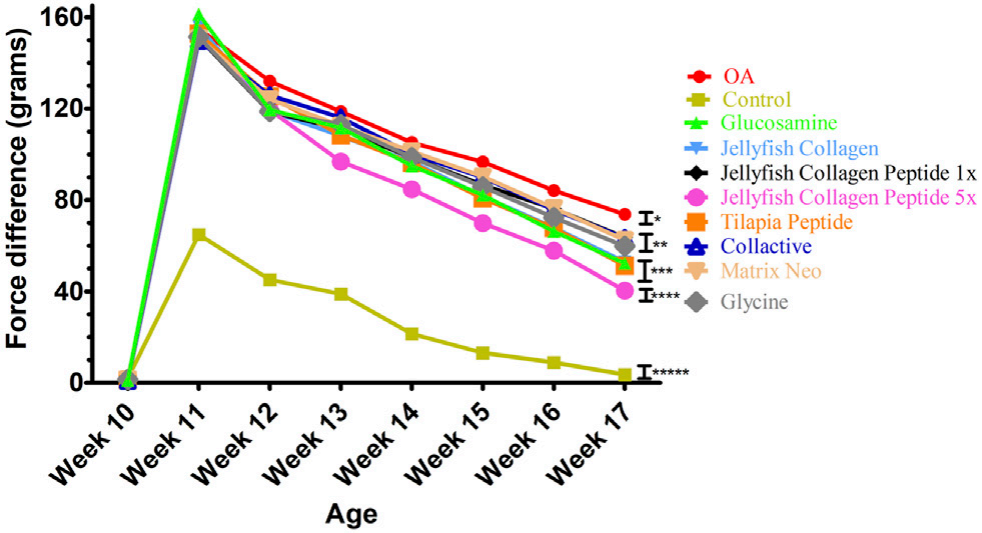
Effects of Collagen, Collagen peptide, Glycine, and glucosamine sulfate treatment on the weight-bearing of the hind limbs in ACLT+MMx surgery-induced OA male rats.

The morphological changes and proteoglycan loss in stained knee joints were then measured, and OA cartilage pathologies were graded using the Osteoarthritis Research Society International (OARSI) guidelines. A rank order is made as follows JCP5 > GS > TC > JC > glycine > matrix > JCP1 > collactive > OA, as shown in Figure 6, and the same trend as OARSI scoring the incapacitance test between rats has the same trend (Figure 7).

## 4 Discussion

Collagen is the most abundant protein in mammals. It is mainly found as part of the extracellular matrix in connective tissues such as cartilage, tendons, ligaments, bones, skin, etc. (Mescher, 2018). Collagen peptides are the product of the enzymatic hydrolysis of native collagen to obtain smaller peptides. The rationality to hydrolyze collagen is not only to improve its solubility but also to improve its bioavailability (Iwai et al., 2005). Collagen peptides are nutraceuticals used in dietary supplements and as food ingredients offering health benefits at different levels. In the administration of native collagen, the mechanism of action is believed to be different from that of collagen peptides, where collagen peptides could function as bioactive peptides or as a source of the specific amino acids needed to build collagen. Due to the low absorption rate of native collagen, the mechanism of action is believed to take place in the gastrointestinal tract. The hypothesis is that, in a similar way to food proteins, native collagen will induce oral tolerance (Park et al., 2009).

In this study, jellyfish collagen was extracted by using papain-assisted extraction. In addition, the FT-IR spectrum of *Rhopilema esculentum* collagen (Figure 1A) was consistent with previously published FT-IR spectra for the same species (Felician et al., 2019). Altogether, the FT-IR spectra indicate a well-maintained secondary structure in the collagen extracted from the *Rhopilema esculentum*. This result demonstrated that the collagen triple helical structure was well-preserved. Jellyfish collagen peptide was obtained by fermentation with *Ganoderma lucidum*. The lower the molecular weight of collagen peptide, the stronger the scavenging activity tends to become (Nurilmala et al., 2020). The fermented collagen peptides tend to show higher antioxidant activities (Table 3), although variable molecular weight distributions also provide striking differences in the antioxidant properties of hydrolyzates (Ketnawa et al., 2016). The specificity of the number and sequence of amino acids in the peptides also affects the hydrolyzate antioxidant activity (Mosquera et al., 2016).

When degenerative musculoskeletal diseases progress, metabolic activities change, and tissue homeostasis is disturbed, resulting in anabolic and catabolic process imbalance. The predominance of catabolic activities effectively results in tissue degeneration in people with osteoarthritis. The articular chondrocytes balance catabolic and anabolic processes in healthy articular cartilage. When catabolism exceeds the capacity of chondrocytes to regenerate, articular cartilage degeneration occurs. If catabolism exceeds the regenerative capacity of the chondrocytes, degeneration of the articular cartilage occurs (Aigner et al., 2006).

In *in vivo* studies, rats treated with collagen and collagen peptide show heavier liver weight than the control. The liver works as the body’s filter, removing toxins from the blood, processing nutrients, and regulating its metabolism (Virović-Jukić and Živković, 2018). Proteins can be converted to lipids *via* amino acid metabolism. Therefore, it can increase the liver weight (Li et al., 2018), which agrees with a recent study from Islam et al., (2018) that a high-protein diet is associated with a physiologically enlarged liver.

When the liver is inflamed or injured, enzymes, including gamma-glutamyl transferase (GGT), leak from the liver cells into the bloodstream. GGT can be utilized in multiple diagnostic and prognostic algorithms or scores for liver disease. It is one of the most powerful predictors of the development of existence of liver disease (Dillon and Miller, 2016). No significant changes were observed in gamma-glutamyl transferase (GGT) levels (data not shown) between groups. Therefore, it may be stated that the ingestion of collagen peptides at 5 mg/b. w. has no risk to the liver. There is no significant difference in the weight of the kidney, which shows there are no kidney problems in the consumption of collagen, collagen peptide, and glycine.

A complex network of factors regulates the anabolic/catabolic balance in articular cartilage, and both sides are impacted during the progression of degeneration. Numerous proinflammatory cytokines, including tumor necrosis factor-α, contribute to the maintenance of the catabolic phenotype during the progression of osteoarthritis (Abramson and Yazici, 2006). TNF-α levels were elevated in untreated groups, and treatment with collagen, collagen peptide, and glycine can significantly decrease the level of TNF-α (Figure 3). TNF-α acts to suppress extracellular matrix synthesis and stimulate the expression of matrix-degrading enzymes (Saklatvala, 1986). By inhibiting TNF-α, the depletion of collagen type II and aggrecan from osteoarthritic cartilage is decreased, and matrix metalloproteinases (MMPs) are also suppressed (Kobayashi et al., 2005), which are enzymes responsible for extracellular matrix degradation.

Nitric oxide (NO) is an inflammatory catabolic mediator that is produced by osteoarthritic chondrocytes (Abramson and Yazici, 2006). NO has been shown to inhibit proteoglycans and collagen biosynthesis in chondrocytes and induce chondrocyte apoptosis (Taskiran et al., 1994; Maneiro, 2004). COX-2 and NO levels in the OA group significantly increase (Figure 3). NO plays a role in the progression of osteoarthritis on various levels and promotes the catabolic processes that result in tissue degeneration. Numerous parallels exist between the nitric oxide (NO) and cyclooxygenase (COX) pathways. NO is the mediator produced by the NO synthase (NOS) pathway, while COX is the enzyme that converts arachidonic acid to prostaglandins (PGs), prostacyclin, and thromboxane A2 (a type of inflammatory mediator) (Cuzzocrea and Salvemini, 2007).

Undenatured type II collagen provided symptom relief, an action attributed to oral tolerance and modulating inflammatory pathways (Tong et al., 2009). Anti-inflammatory cytokines released by Treg cells specific for type II collagen are critical for the cells’ ability to promote oral tolerance (Asnagli et al., 2014). IL-10’s anti-inflammatory properties protect against tumor necrosis factor-alpha–induced damage (Müller et al., 2008). Therefore, our results confirm that treatment with collagen can significantly reduce levels of TNF-α (Figure 3).

Collagen peptides can inhibit the activation of JNK and ERK pathways (Subhan et al., 2017). In the present study, collagen peptides demonstrated an anti-inflammatory capability (Figure 3) with suppression of TNF-α, COX-2, and NO levels. Nitric oxide may be reduced and inhibited due to the ability of collagen peptides’ –OH and–NH2 groups to bind to nitric oxide radicals (Zhu et al., 2014). Glycine has been reported to interfere with NF-kB’s TNF-α-mediated activation (Hasegawa et al., 2012). Glycine may inhibit the phosphorylation or ubiquitin-dependent degradation of IkBs or it could speed up the removal of active forms of NF-kB from the nucleus or it could be a combination of these mechanisms (Beinke and Ley, 2004; Bassères and Baldwin, 2006). Therefore, glycine can significantly reduce the levels of COX-2 and NO (Figure 3).

MMP-13 is by far the most investigated metalloproteinase in cartilage pathology (Rose and Kooyman, 2016). Since MMP-13 plays a crucial role in OA, it has become a hot spot that researchers are paying attention to. CTX-II is a C-terminal peptide that is produced by the concerted action of matrix metalloproteinase (MMPs) on fibrillar type II collagen. It is considered to be a biomarker of cartilage degradation because of its association with the degradation of cartilage (De Ceuninck et al., 2003; Rousseau and Delmas, 2007). Its level was found to be correlated with cartilage loss in osteoarthritis animal models (De Ceuninck et al., 2003). We speculate that decreased cytokine concentrations result in decreased MMP13 expression. The positive behavioral effects of collagen, collagen peptide, and glycine may be related to prevention of articular damage since CTX-II levels were reduced in the plasma of treated rats (Figure 4). (Ohnishi et al., 2013) also discovered that rabbits administered fish collagen peptides were protected from induced cartilage degradation (OA) and had some protective effects on cartilage structure and integrity. These results provide further evidence that collagen, collagen peptide, and glycine have a therapeutic effect on OA progression.

We have used Safranin-O staining in OA cartilage to confirm that collagen, collagen peptide, and glycine can recover cartilage metabolism’s homeostasis and suppress OA development. The articular cartilage in the control group was well-stained with Safranin O (which stains PG red) with no apparent loss of staining intensity, indicating that the matrix’s PG content was normal. The osteoarthritis group’s articular cartilage significantly reduced Safranin O staining intensity in the non-calcified region and a slight reduction in the calcified region, indicating a significant decrease in the matrix’s PG content. In addition, the articular cartilage’s surface was fibrillated.

The treated group shows a recovery of cartilage and less fibrillation in every group, especially in the Rhopilema collagen group, glycine group, glucosamine group, tilapia collagen peptide, and Rhopilema collagen 5x dose group. It showed that cartilage of the treated group had better conservation than that of the untreated group (Figure 5). When it comes to cartilage, proteoglycan is an important component that protects chondrocytes and is responsible for the structural integrity of cartilage tissue. As a result, increasing its concentration is important for the prevention of cartilage deterioration (Knudson and Knudson, 2001; Chen et al., 2014).

Decreased level of secreted MMP13 (Figure 4) observed in collagen-treated OA rats might directly contribute to increased proteoglycan content (Figure 5). In addition, collagen significantly decreased the number of apoptotic chondrocytes and improved the status of the cartilage matrix. In agreement with (Geahchan et al., 2022), collagen peptide ameliorates osteoarthritis progression by promoting extracellular matrix synthesis, promoting the formation of type II collagen (Figure 4). This effect could very well be due to the absorption of functional peptides such as Gly-Pro-Hyp and Pro-Hyp by the body following oral administration of collagen peptides. Since Gly-Pro-Hyp and Pro-Hyp are the primary peptides that make up type I and II collagen, Gly-Pro-Hyp and Pro-Hyp may activate cells found in collagen-rich tissues.

In addition, both Gly-Pro-Hyp and Pro-Hyp can operate as inducers of ECM production in cells (Min, 2017). Different molecule sizes could also account for discrepancies in the observed cell proliferation stimulation activity (Lu and Zhao, 2018). Treatment with glycine shows presence of a smooth surface and recovered chondrocyte. However, with only glycine, maybe there are other limiter amino acids to rebuild the collagen in the cartilage, making collagen type II production lower in glycine-treated rats. Glycine should always be consumed with a high-protein diet to prevent its metabolism from synthesizing other non-essential amino acids that may be deficient in a low-protein diet, thereby deviating glycine from its intended function and preventing it from producing the desired effect (Meléndez-Hevia et al., 2021).

The morphological changes and proteoglycan loss in stained knee joints were then measured, and OA cartilage pathologies were graded using the Osteoarthritis Research Society International (OARSI) guidelines. A rank order is made as JCP5 > GS > TC > JC > glycine > matrix > JCP1 > collactive > OA, and the same trend as OARSI scoring the incapacitance test between rats have the same trend.

## 5 Conclusion

The collagen from jellyfish was successfully extracted using papain-assisted acid extraction, and the collagen peptide from jellyfish was successfully produced using *Ganoderma lucidum* fermentation. As a result of their ability to inhibit the production of proinflammatory cytokines such as COX-2, NO, MMP-13, and CTX-II while also increasing cartilage thickness, collagen and collagen peptides can be used as an osteoarthritis treatment. Jellyfish collagen peptide at a dose of 5 mg/kg b. w., compared to other collagen peptides, has the greatest potential for treating osteoarthritis (OA) in the knee because of its protective and cartilage-regenerating properties.

## Data Availability

The original contributions presented in the study are included in the article/Supplementary Material; further inquiries can be directed to the corresponding authors.

## Supplementary Material

Supplementary Data 1 Graphical Abstract.pptx

Supplementary Data 2 Photos of the materials.jpg
