# Supplementary figures and images for: Effect of Different Collagen on Anterior Cruciate Ligament Transection and Medial Meniscectomy-Induced Osteoarthritis Male Rats
Source: Front Bioeng Biotechnol. 2022 Jul 5;10:917474. doi: 10.3389/fbioe.2022.917474 (PMC9295145; doi:10.3389/fbioe.2022.917474)

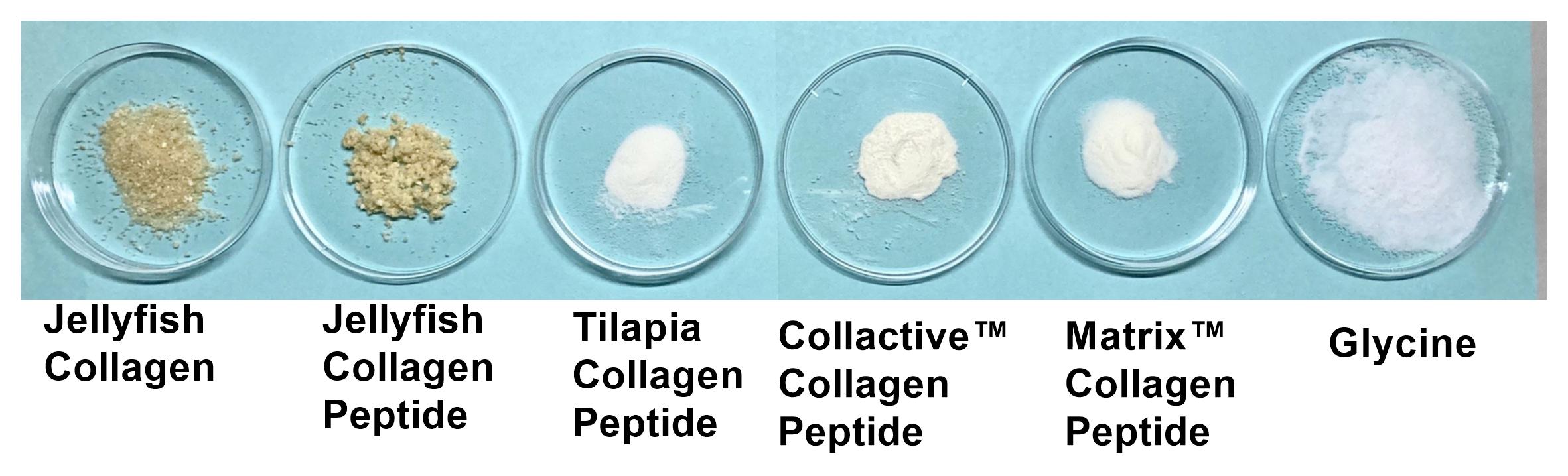

Supplement: Supplementary file 1 [file Image1.JPEG]
